# Syndecan‐4 influences mammalian myoblast proliferation by modulating myostatin signalling and G1/S transition

**DOI:** 10.1002/1873-3468.13227

**Published:** 2018-09-07

**Authors:** Aniko Keller‐Pinter, Kitti Szabo, Tamas Kocsis, Ferenc Deak, Imre Ocsovszki, Agnes Zvara, Laszlo Puskas, Laszlo Szilak, Laszlo Dux

**Affiliations:** ^1^ Department of Biochemistry Faculty of Medicine University of Szeged Hungary; ^2^ Drem Ltd. Budapest Hungary; ^3^ Biological Research Centre Hungarian Academy of Sciences Szeged Hungary; ^4^ Szilak Laboratories Bioinformatics & Molecule‐Design Ltd. Szeged Hungary

**Keywords:** myoblast, myostatin, syndecan‐4

## Abstract

Myostatin, a TGF‐β superfamily member, is a negative regulator of muscle growth. Here we describe how myostatin activity is regulated by syndecan‐4, a ubiquitous transmembrane heparan sulfate proteoglycan. During muscle regeneration the levels of both syndecan‐4 and promyostatin decline gradually after a sharp increase, concurrently with the release of mature myostatin. Promyostatin and syndecan‐4 co‐immunoprecipitate, and the interaction is heparinase‐sensitive. ShRNA‐mediated silencing of syndecan‐4 reduces C2C12 myoblast proliferation *via* blocking the progression from G1‐ to S‐phase of the cell cycle, which is accompanied by elevated levels of myostatin and p21(Waf1/Cip1), and decreases in cyclin E and cyclin D1 expression. Our results suggest that syndecan‐4 functions as a reservoir for promyostatin regulating the local bioavailability of mature myostatin.

## Abbreviations

**EGFR**, epithelial growth factor receptor

**FGF**, fibroblast growth factor

**GAG**, glycosaminoglycan

**GDF8**, growth‐ and differentiation factor 8

**HER2**, human epidermal growth factor receptor 2

**HGF**, hepatocyte growth factor

**SDC**, syndecan

**TGF‐β**, transforming growth factor beta

Skeletal muscle is constantly renewed in response to injury, exercise, or muscle diseases. A population of resident stem cells (satellite cells) accounts for skeletal muscle plasticity, maintenance and regeneration 1, 2, 3. The muscle progenitor satellite cells are mitotically and physiologically quiescent in healthy muscle; they are stimulated by local damage to proliferate extensively and form myoblasts that will subsequently differentiate and fuse to form muscle fibres. By understanding the process of skeletal muscle regeneration we might have the possibility to improve it following sport injuries or during aging. Several studies described that proteoglycans and other components of the extracellular matrix are involved in tissue regeneration and skeletal muscle differentiation 4, 5, 6. The crucial roles of syndecan (SDC) proteoglycans have been shown in the regeneration of the skin, vasculature and skeletal muscle 6.

Syndecans (SDCs) constitute a family of four transmembrane heparan sulfate proteoglycans in mammals 7, 8, 9. They are composed of three distinct domains, an N‐terminal variable extracellular domain with glycosaminoglycan attachment sites, a single conserved transmembrane domain and a short C‐terminal cytoplasmic domain with two conserved regions flanking a variable region unique for each SDC. The extracellular domains are variable between the SDC family members, whereas the transmembrane and cytoplasmic domains are highly conserved. SDC1 is mainly expressed in epithelial and plasma cells, SDC2 is mostly found in mesenchymal cells (fibroblasts and smooth muscle) and SDC3 is abundant in neural tissues and developing musculoskeletal tissues. In contrast to other SDCs, SDC4 is expressed ubiquitously 7. Among the different glycosaminoglycan (GAG) chains, which are attached to the protein core of SDCs, both heparan sulfate and chondroitin sulfate chains are represented in SDC1 and SDC3, but only heparan sulfates are attached to the SDC2 and SDC4 ectodomains.

Heparan sulfates on proteoglycans can either negatively or positively regulate growth factor function by participating as co‐receptors, reservoirs for storage or transporters 10. Cell surface heparan sulfate proteoglycans can recruit soluble ligands, thereby increasing their local concentration, and they can also modulate ligand receptor encounters 7, 11, or can protect the growth factors from proteolytic inactivation 12. The ectodomains of SDCs mediate several cell–cell and cell–matrix interactions *via* the GAG chains. The extracellular domains interact with matrix proteins and numerous growth factors; therefore, SDCs are usually considered as co‐receptors of the primary signalling receptors. Interactions of HER2 (human epidermal growth factor receptor 2) and EGFR (epithelial growth factor receptor) with SDC1 and SDC4 respectively, have been reported 13, and SDC1 was identified as a co‐receptor for HGF (hepatocyte growth factor) 14. Signalling by FGF (fibroblast growth factor) family members and HGF is regulated by heparan sulfates 15, 16, and both HGF and the members of the FGF family have been implicated in satellite cell activation and skeletal muscle differentiation 10.

One of the cell surface markers of quiescent and proliferating muscle progenitor satellite cells is SDC4 17. It was reported that SDC4−/− mice are unable to regenerate damaged muscle and explanted satellite cells were deficient in activation, proliferation and MyoD expression 18. The ubiquitously expressed SDC4 has an important role in outside‐in and inside‐out signalling events in different cell types by, for example influencing cell‐matrix adhesion, endocytosis, exosome biogenesis, cytokinesis and regulating the activity of Rac1 GTPase and the level of intracellular calcium 7, 8, 9, 19.

Myostatin, also known as growth‐ and differentiation factor 8 (GDF8), belongs to the TGF‐beta (transforming growth factor beta) superfamily. It is synthesized as a precursor protein, promyostatin, which is cleaved into N‐terminal propeptide and C‐terminal active myostatin fragments by the furin family of proprotein convertases 20, 21. This cleavage can occur in the Golgi network or in the extracellular matrix. Anderson and colleagues identified an extracellular promyostatin pool, which can be activated by furin, thus localizing myostatin activity through extracellular localization of promyostatin maturation 22. The propeptides can still associate with myostatin dimer *via* noncovalent bonds to form an inactive latent complex which sequesters functional myostatin by preventing its binding to the receptor 21. The members of the bone morphogenetic protein‐1/tolloid (BMP‐1/TLD) family of metalloproteinases are involved in activating this latent myostatin *in vivo* 23. The mature myostatin dimer acts through activin type II receptor ActRIIB and to a lesser extent ActRIIA 21. The signalling involves the phosphorylation of Smad2/3 transcription factors 24, 25, and the inhibition of the PI3K/Akt pathway 26, 27. Myostatin was shown to up‐regulate the expression of p21(Waf1/Cip1), thus preventing the progression of myoblasts from the G1‐ to S‐phase of the cell cycle 28. It also inhibits myoblast differentiation by down‐regulating the synthesis and activity of the muscle regulatory factor MyoD 24.

SDC4 has an essential role in skeletal muscle development and regeneration 18; however, its specific role in mammalian myoblast (activated satellite cell) proliferation has not been studied yet. In the present study we found that SDC4 regulates the proliferation of myoblasts, and silencing of SDC4 decreases the progression of the cell cycle from G1‐ to S‐phase. Furthermore, we have shown that SDC4 interacts with promyostatin in a heparan sulfate‐dependent manner and influences the level of mature myostatin. Our results suggest that SDC4 may regulate the local bioavailability of mature myostatin by serving as a reservoir for promyostatin and subsequently inhibiting the formation of active myostatin.

## Materials and methods

### Animal model

For the regeneration model of skeletal muscle, the necrosis of soleus muscle (m. soleus) of male Wistar rats (300–320 g) was induced by the snake venom notexin (from *Notechis scutatus scutatus*; Sigma‐Aldrich, St. Louis, MO, USA) under chloral hydrate anaesthesia as described previously 29. Briefly, 20 μg notexin in 200 μL of 0.9% NaCl solution was injected along the whole length of the muscle. The muscles were removed under anaesthesia on days 0, 1, 3, 4, 5, 7, 10 and 14 after injury (*n* = 4 in each group). All animal experiments were conducted under the approval of the Animal Health Care and Control Institute, Csongrad County, Hungary.

### Cell culture and plasmids

C2C12 mouse myoblast cells (ATCC, Manassas, VA, USA) were cultured in Dulbecco's modified Eagle's medium (4.5 g·L^−1^ glucose with glutamine; Lonza, Basel, Switzerland) containing 50 μg·mL^−1^ gentamycin (Lonza) and supplemented with 20% fetal bovine serum (Gibco, Thermo Fisher Scientific, Waltham, MA, USA). Differentiation was induced by shifting the confluent cultures to medium containing 2% horse serum (Sigma‐Aldrich). For SDC4 silencing the C2C12 cells were stably transfected with plasmids expressing short hairpin RNAs (shRNA) targeting mouse SDC4 (shSDC4#1 and shSDC#2), a scrambled target sequence, or the empty pRS vector using X‐tremeGENE transfection reagent (Roche, Basel, Switzerland). The transfected populations were selected in medium supplemented with 4 μg·mL^−1^ puromycin (Sigma‐Aldrich). The plasmids were obtained from OriGene (TR513122; Rockville, MD, USA) and targeted the sequences 5′‐GAA CTG GAA GAG AAT GAG GTC ATT CCT AA‐3′ (shSDC4#1), 5′‐GCG GCG TGG TAG GCA TCC TCT TTG CCG TT‐3′ (shSDC4#2) and 5′‐GCA CTA CCA GAG CTA ACT CAG ATA GTA CT‐3′ (scrambled).

### Staining, microscopy

Frozen sections (10 μm) of control and regenerating soleus muscles were fixed in acetone for 5 min and were stained by haematoxylin (0.1%) and eosin (1%). Photos were taken with 20× objective using a Nikon Labophot‐2 microscope equipped with Olympus DP71 camera. Cell cultures were analysed with a Leica DMi1 inverted microscope.

### Protein isolation and western blotting

Rat soleus muscles were homogenized in 50 mm Tris‐HCl pH 7.6, 100 mm NaCl, 10 mm EDTA buffer containing 1 mm Na‐fluoride, 1 mm Na_3_VO_4_ and protease inhibitor cocktail (Sigma‐Aldrich). C2C12 cells were harvested in RIPA buffer (20 mm Tris‐HCl pH 7.5, 150 mm NaCl, 1 mm Na_2_EDTA, 1 mm EGTA, 1% NP‐40, 1% sodium deoxycholate, 2.5 mm sodium pyrophosphate, 1 mm b‐glycerophosphate, 1 mm Na_3_VO_4_, 1 μg·mL^−1^ leupeptin; Cell Signaling Technology, Danvers, MA, USA; #9806) supplemented with 1 mm Na‐fluoride, and protease inhibitor cocktail (Sigma‐Aldrich). After centrifugation of the samples at 160 000 ***g*** for 5 min at 4 °C to eliminate cellular debris the supernatants were separated by SDS/PAGE, blotted to nitrocellulose or poly(vinylidene difluoride) membrane. After blocking, membranes were incubated with antibodies including goat anti‐SDC4 (sc‐9499; Santa Cruz Biotechnology, Santa Cruz, CA, USA), rabbit anti‐SDC4 (36‐3100; Zymed/Thermo Fisher Scientific), rabbit anti‐myostatin (AB3239; Chemicon/Merck, Kenilworth, NJ, USA or AB3239‐I; Merck Millipore; Billerica, MA, USA), both recognizing the C‐terminal part of the protein, anti‐phospho‐Smad2^Ser465/467^ (44‐244G; Invitrogen, Carlsbad, CA, USA), mouse anti‐GAPDH (#2118; Cell Signaling Technology), rabbit anti‐myoD (sc‐304), mouse anti‐p21 (sc‐6246), mouse anti‐cyclin D1 (sc‐6281) and rabbit anti‐cyclin E (sc‐481; all from Santa Cruz Biotechnology) primary antibodies, followed by incubation with the appropriate horse‐radish peroxidase‐conjugated anti‐IgG secondary antibodies [anti‐mouse (P0161), anti‐rabbit (P0448), anti‐goat (P0160)] from DAKO (Glostrup, Denmark). Peroxidase activity was visualized by the ECL procedure (Advansta, Menlo Park, CA, USA). Quantification of signal intensity was performed by quantity one software (Bio‐Rad, Hercules, CA, USA).

### Co‐immunoprecipitation and heparinase digestion

Homogenates were pre‐cleared with Protein A/G beads in an effort to reduce the possibility of nonspecific binding of proteins to the beads. Afterwards the supernatants were incubated overnight with the antibody of interest followed by incubation with immobilized Protein A/G (Pierce, Rockford, IL, USA) for 2 h. Protein A/G slurry was collected by pulse centrifugation, followed by washing three times with immunoprecipitation buffer (25 mm Tris, 150 mm NaCl, pH 7.2). The immunocomplex was eluted with 0.2 m glycin (pH 2.5). The eluted immunocomplex was subjected to SDS/PAGE, followed by immunoblotting with the appropriate antibodies.

To test the role of heparan sulfate chains, the samples were incubated with anti‐SDC4 antibody overnight. After incubation with Protein A/G, the protein A/G slurry was washed two times with immunoprecipitation buffer and once with heparinase buffer (50 mm HEPES pH 6.5, 50 mm NaOAc, 150 mm NaCl, 5 mm CaCl_2_). The immunoprecipitate was resuspended in heparinase buffer and digested with 0.4 mU heparinase II enzyme (Sigma‐Aldrich) for 3 h at 37 °C. After digestion both the supernatant and the eluted immunocomplex were subjected to SDS/PAGE.

### QRT‐PCR analysis

For qRT‐PCR, total RNA was isolated from C2C12 cell lines and reverse transcribed (three samples for each cell line). TaqMan probe sets [SDC1: Mm01275869_m1, SDC2: Mm04207492_m1, SDC3: Mm01179833_m1, SDC4: Mm00488527_m1, glypican‐1 (Gpc1): Mm01290371_m1, perlecan (Hspg2): Mm01181173_g1, myostatin: Mm00440328_m1, HPRT (hypoxanthine‐guanine phosphoribosyltransferase): Mm03024075_m1; all from ThermoFisher Scientific] and the TaqMan Master Mix (Roche) were used with the following program: 10 min at 95 °C, 45 cycles of 95 °C for 15 s and 60 °C for 1 min. Individual threshold cycle (*C*_t_) values were normalized to the *C*_t_ values of HPRT. Relative gene expression levels are presented as log_2_ ratios.

### Cell proliferation assay

Equal number of cells of the nontransfected and transfected cell lines were plated and grown in proliferation media. CellTiter‐Glo assays (Promega, Madison, WI, USA) were performed according to the manufacturer's instructions at 12, 24 and 36 h after seeding. The luminescence was measured on FLUOstar Optima plate reader (BMG Labtech, Ortenberg, Germany).

### Cell cycle analysis

The collected cells were washed once with PBS, resuspended in PBS, and fixed by addition of ice‐cold 96% ethanol to 70% final concentration. The fixed cells were pelleted by centrifugation (at 350 ***g***, 5 min, 4 °C), then resuspended in PBS containing 50 μg·mL^−1^ RNaseA (#EN0531; Thermo Fisher Scientific). After 30 min incubation at 37 °C propidium iodide (Sigma‐Aldrich) was added to 1 μg·mL^−1^ final concentration. Flow cytometry was performed with a Partec FlowMax 3.0 flow cytometer (Sysmex Partec GmbH, Görlitz, Germany), and the data were analysed using flowjo software (FlowJo LLC, Ashland, OR, USA).

### Statistical analysis

Statistical evaluations were performed by one‐way ANOVA and Newman–Keuls post‐test (GraphPad Software Inc., La Jolla, CA, USA). All data are presented as means ± SEM.

## Results

### The expression of syndecan‐4 and myostatin during *in vivo* and *in vitro* myoblast differentiation

**Figure 1:**
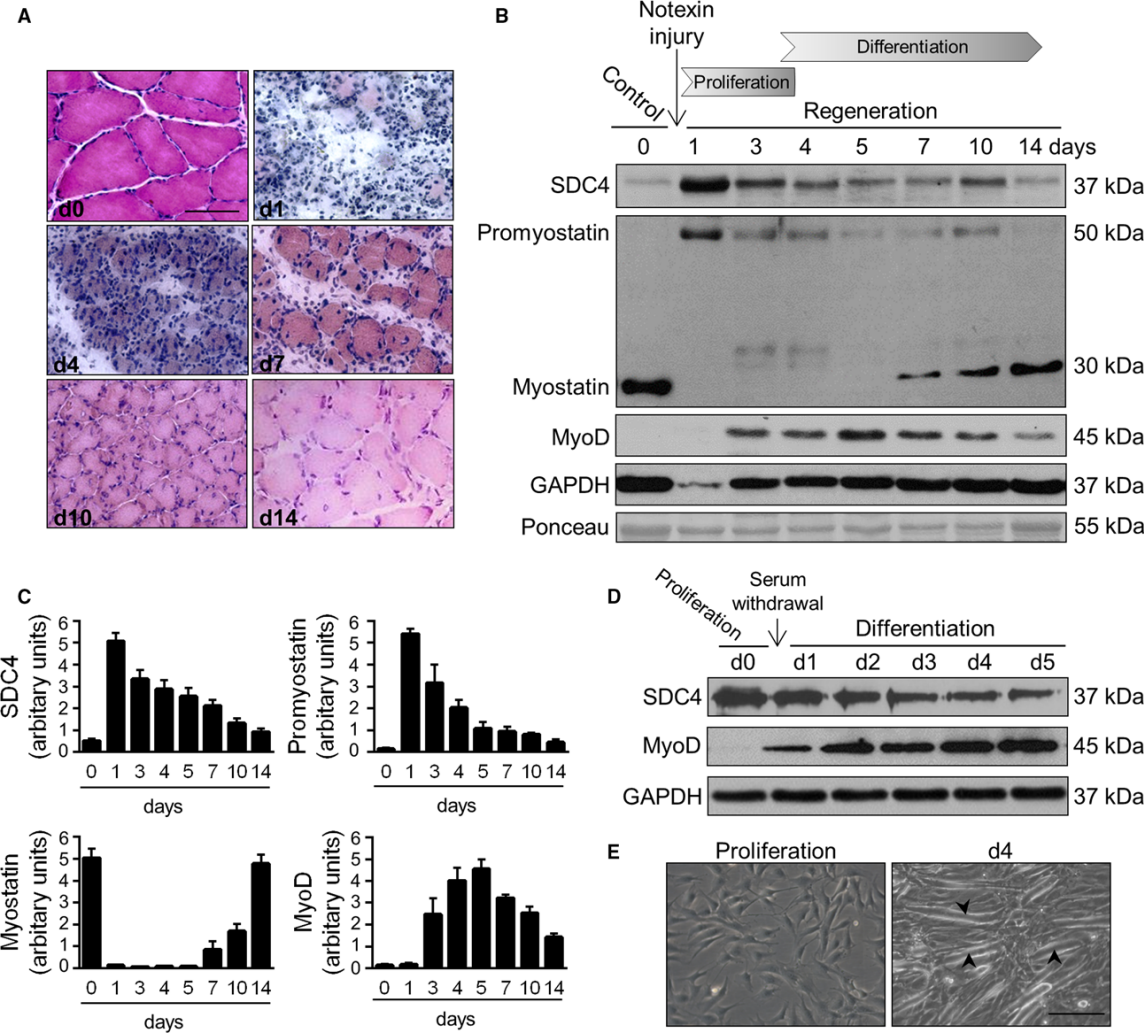
Expression of SDC4 and myostatin during skeletal muscle regeneration. (A) Representative haematoxylin and eosin‐stained sections of control and regenerating soleus muscle of the rat on different days after notexin injection. Bar: 50 μm. (B) Aliquots of extracts containing equivalent amounts of protein obtained from m. soleus on different days after notexin induced injury were subjected to SDS/PAGE, and immunoblotted with anti‐SDC4, anti‐myostatin (AB3239‐I), anti‐MyoD and anti‐GAPDH antibodies. Representative immunoblots are shown. GAPDH level is decreased after the injury in the necrotic muscle. Representative Ponceau staining of the membrane is presented. (C) Quantification of results, data are reported as means ± SEM (*n* = 4 independent experiments at each time point). (D) Expression of SDC4 during the differentiation of C2C12 myoblasts (0–5 days). GAPDH shows the equal loading of the samples. (E) Representative images of proliferating and differentiating (day 4) C2C12 cells. Arrowheads show the formation of myotubes. Bar: 200 μm.

Muscle regeneration can be artificially induced by injecting the snake venom notexin. It rapidly induces myonecrosis and, because it does not affect the muscle progenitor satellite cells, a subsequent regeneration of the tissue 30. To monitor the process of regeneration the cryo‐sections of regenerating m. soleus of the rat were stained with haematoxylin and eosin (Fig. 1). In the first 3 days abundant inflammatory cells and proliferating myoblasts were observed between the necrotic fibres. By days 4–5 regenerating small calibre myofibres appeared with centrally located nuclei, on day 7 most of the myofibres had the nuclei in central position, but their diameters were highly heterogeneous. By day 14 the muscle restored its normal morphology with a persistence of central nuclei and a slightly increased interstitial space (Fig. 1A).

To examine the expression of proteins during regeneration we analysed the homogenates of soleus muscle on different days after notexin injection (Fig. 1B,C). Western blot experiments showed a transient upregulation of SDC4 expression during the proliferation phase, and simultaneous a low level of mature myostatin and high level of promyostatin. The expression of SDC4 markedly increased on day 1, but gradually decreased to the level of the untreated control sample by day 14. During the proliferation phase we observed little or no mature myostatin, and during the differentiation phase the expression increased (Fig. 1B,C). In contrast, the expression of precursor promyostatin changed inversely with that of myostatin, indicating the enhanced proteolytic cleavage of promyostatin during the regeneration. By day 14 the expression levels of promyostatin and SDC4 were similar to those in untreated muscle (Fig. 1B,C). The regeneration process was monitored by the expression of the muscle regulatory factor MyoD (Fig. 1B,C).

An excellent *in vitro* model exists to study muscle differentiation, since shifting mouse C2C12 myoblasts from growth medium to low‐serum fusion medium induces the formation of multinucleated, myosin expressing myotubes 31. We transferred proliferating C2C12 cells to differentiation medium, and monitored the expression pattern of SDC4. Western blot analysis showed increased SDC4 expression in proliferating myoblasts, and the level of SDC4 decreased during the differentiation (Fig. 1D), similar to what was seen in the *in vivo* model. Representative phase contrast images show myotube formation during the differentiation (Fig. 1E).

### SDC4 interacts with promyostatin in a heparan sulfate‐dependent manner

**Figure 2:**
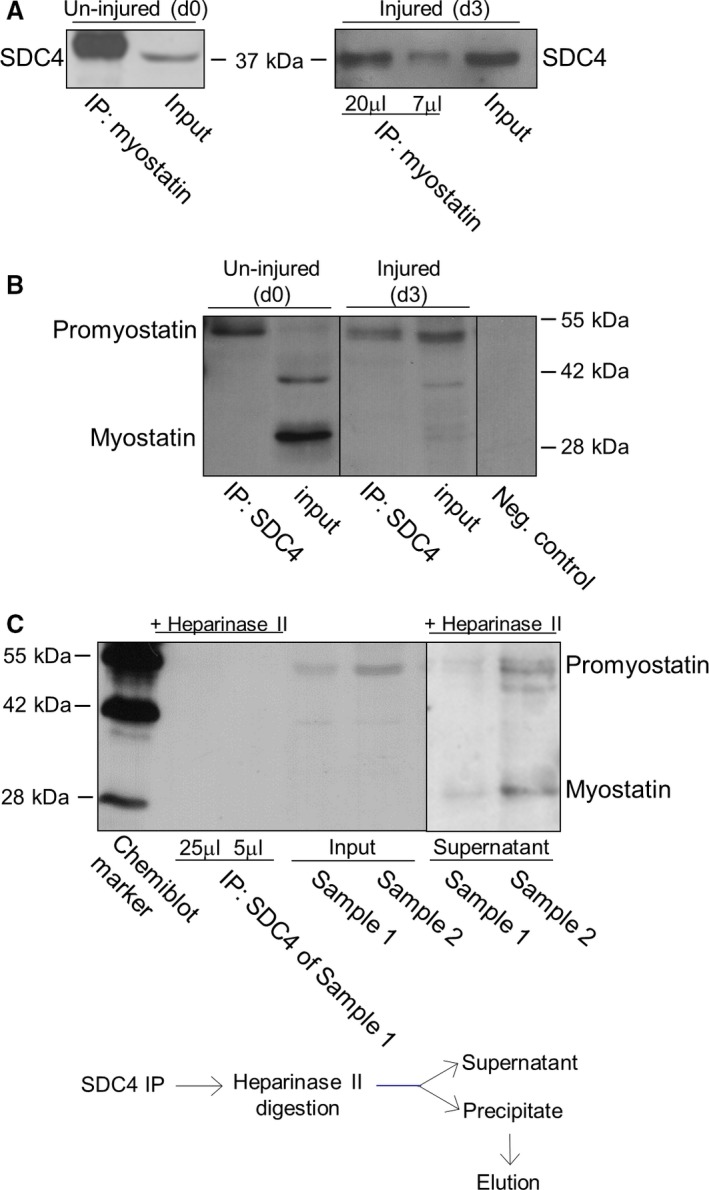
Characterization of SDC4–promyostatin interaction. (A) Co‐immunoprecipitations (co‐IPs) were carried out with rabbit antiserum to the C‐terminal part of myostatin (AB3239) in un‐injured (control, day 0, d0) and injured (3 days after notexin injury, d3) soleus muscle homogenates. Different volumes (20 and 7 μL) of the eluted immunocomplex were loaded in case of the injured sample. The blots were reacted with antibodies to SDC4 raised in goat. Myostatin co‐immunoprecipitated SDC4 in both cases. Input lanes represent the total homogenates; 10% of the total protein amount used in co‐IP was loaded. (B) Co‐IP assays were performed with anti‐SDC4 antibodies, and the blots were reacted with anti‐myostatin antisera (AB3239). The negative control was incubated only with the secondary antibody. For the input lanes, 10% of the total protein amount used in co‐IP was loaded. The additional band at ~ 42 kDa in d0 input can be a processing intermediate of promyostatin. (C) Heparan sulfate chains were digested in injured samples (d3; sample 1 and 2) with heparinase II enzyme following immunoprecipitation with goat anti‐SDC4 antibody. Different amounts (25 and 5 μL) of the eluted volume of the heparinase II digested immunoprecipitate of sample 1 were loaded. We could not detect promyostatin in the immunoprecipitate after heparinase digestion. For the input lanes, 7.5% of the total protein amount used in co‐IPs were loaded. Note, that both promyostatin and mature myostatin were detected in the supernatant (digestion buffer) after heparinase digestion.

Since proteoglycans can bind and serve as a reservoir for numerous growth factors this raised the question whether SDC4 can bind myostatin. We performed co‐immunoprecipitation (co‐IP) assays in un‐injured (control, day 0) and injured (3 days after notexin injury) m. soleus samples to monitor the potential interaction. Anti‐myostatin antibody co‐immunoprecipitated SDC4 (Fig. 2A), and SDC4 co‐immunoprecipitated with promyostatin (Fig. 2B) in both un‐injured and injured soleus muscle samples. SDC4 mainly interacted with promyostatin, the mature myostatin was not detectable in the immunocomplex. Importantly, heparinase II treatment following SDC4 co‐IP abolished the interaction of SDC4 with promyostatin (Fig. 2C) indicating the role of heparan‐sulfate chains in the interaction. Both promyostatin and mature myostatin were detected in the supernatant (digestion buffer) collected after heparinase digestion of the immunocomplex (Fig. 2C).

### SDC4 knockdown influences the levels of heparane sulfate proteoglycans and myostatin

**Figure 3:**
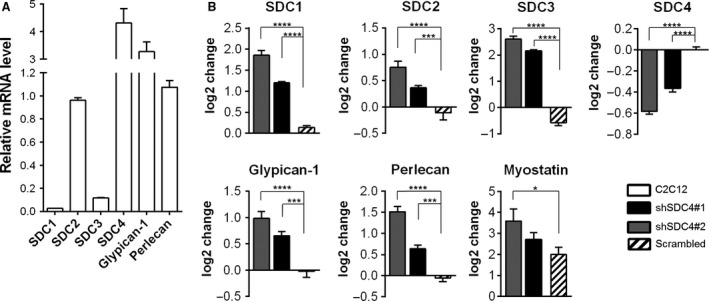
Gene expression of heparan sulfate proteoglycans in C2C12 cells, the effect of SDC4 silencing. (A) qRT‐PCR experiments were performed to analyse the transcript levels of heparan sulfate proteoglycans (SDC1, SDC2, SDC3, SDC4, glypican‐1 and perlecan) in C2C12 cells. Relative mRNA levels are shown, individual threshold cycle (*C*_t_) values were normalized to the *C*_t_ values of HPRT. (B) Effect of SDC4 silencing on the transcript levels of heparan sulfate proteoglycans and myostatin. The log_2_ change values compared to empty vector‐transfected cells are shown. Data are reported as means ± SEM (*n* = 3 independent experiments/each cell line). Data are reported as means ± SEM; *P < 0.05, ***P < 0.001, ****P < 0.0001.

We performed qPCR assays to monitor the gene expression of SDC family members and other heparan sulfate proteoglycans in C2C12 myoblast cells. C2C12 cells express all members of the SDC family; SDC4 is the most abundant, and glypican‐1 and perlecan are also present (Fig. 3A). Silencing of SDC4 upregulated the levels of SDC3 and SDC1, and slightly increased the amount of SDC2 transcripts (Fig. 3B). The heparan sulfate proteoglycan glypican‐1 and perlecan showed weak upregulation following SDC4 silencing. Furthermore, the transcript levels of the myostatin gene were also measured. The level of myostatin mRNA increased in SDC4 knockdown cells, which was significant in shSDC4#1 cell line (Fig. 3B).

**Figure 4:**
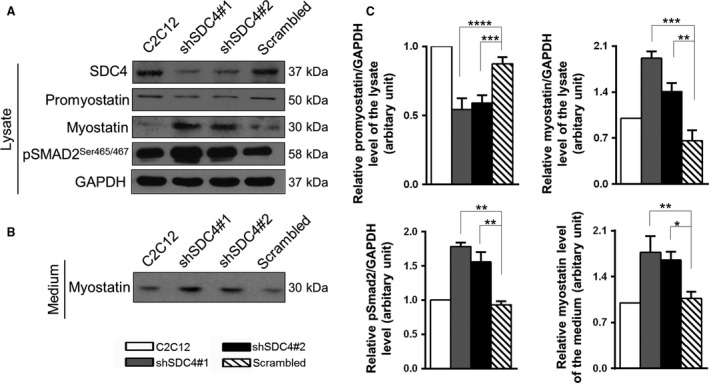
SDC4 influences the level of myostatin. (A) Representative western blot experiments show the levels of SDC4, promyostatin, myostatin and phospho‐pSMAD2^Ser465/467^ in nontransfected C2C12 myoblasts and in cell lines stably expressing shRNA against SDC4 (shSDC4#1, shSDC4#2) or scrambled shRNA. GAPDH shows the equal loading of the samples. (B) Cell culture media of the cells shown in panel A were collected and subjected to SDS/PAGE, followed by immunoblotting with anti‐myostatin antibody. (C) Quantification of the results is reported as means ± SEM (*n* = 3–5 independent experiments); *P < 0.05, **P < 0.01, ***P < 0.001, ****P < 0.0001.

We have found that SDC4 interacts with promyostatin; therefore, we tested the levels of promyostatin and myostatin proteins in nontransfected C2C12 cells and in cell lines stably transfected with plasmids expressing shRNA against SDC4 (shSDC4#1, shSDC4#2) or scrambled shRNA. We found that SDC4 silencing increased the level of mature myostatin of the cells, and decreased the amount of precursor promyostatin (Fig. 4A); furthermore, increased the level of phospho‐pSMAD2^Ser465/467^ (Fig. 4A,C) indicating enhanced myostatin signalling. Importantly, we observed a significantly increased myostatin content in the cell culture medium of SDC4 silenced cells (Fig. 4B,C) consistent with the increased myostatin level of the cells.

### Silencing of SDC4 decreases the proliferation rate of C2C12 myoblasts by decreasing the progression from G1‐ to S‐phase of the cell cycle

**Figure 5:**
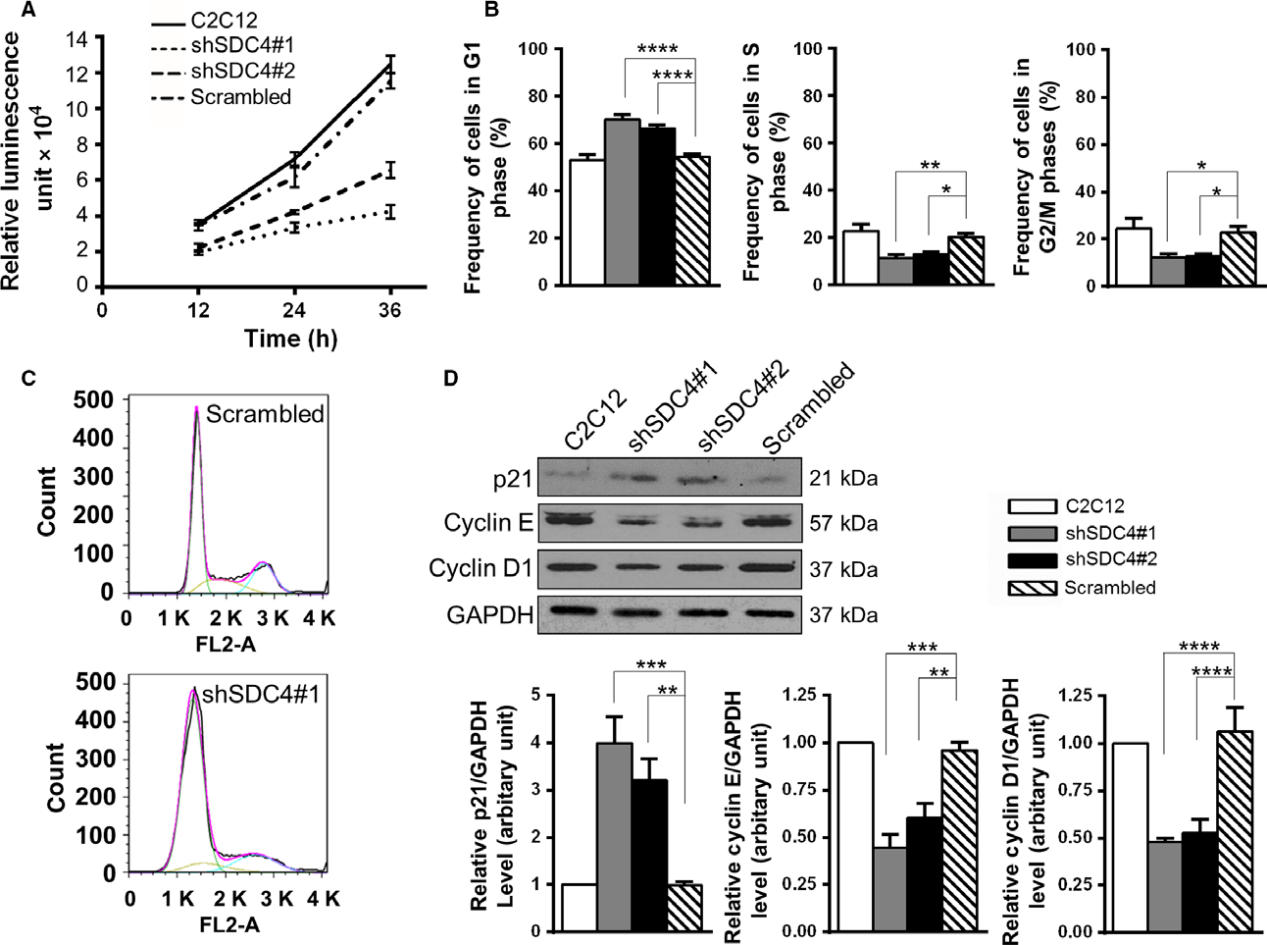
SDC4 knockdown reduces the proliferation rate of C2C12 myoblasts by decreasing the progression from G1‐ to S‐phase of the cell cycle. (A) Proliferation of the nontransfected C2C12 cells, and cell lines stably transfected with vectors expressing shRNA against SDC4 or scrambled shRNA was monitored by CellTiter‐Glo assay for 36 h (*n* = 3 independent experiments/cell line at each time point). (B) Quantification of the frequency of the cells in G1, S or G2/M phases of the cell cycle. Data are reported as means ± SEM,* n* = 8–15 independent experiments/cell line; 20 000 cells/cell line were counted in each experiment; *P < 0.05, **P < 0.01, ****P < 0.0001. (C) Representative images of the cell cycle analysis of scrambled and shSDC4#1 cells with the fitted curve in pink and respective cell cycle phase terms in green (G1), yellow (S) and blue (G2/M). (D) Representative western blot images show the expression levels of p21, cyclin E and cyclin D1 proteins in the different cell lines. Quantifications of results are shown (*n* = 4–10 independent experiments). Data are reported as means ± SEM; **P < 0.01, ***P < 0.001, ****P < 0.0001.

SDC4 can bind growth factors, and here we showed that it can bind promyostatin. Therefore, we tested the effect of SDC4 knockdown on myoblast proliferation. Decreased proliferation of SDC4 silenced cells was observed compared to nontransfected cells and myoblasts expressing a scrambled sequence (Fig. 5A). The proliferation rate of shSDC4#1‐transfected cells was lower than that of the shSDC4#2 line in accordance with the lower SDC4 level of shSDC4#1 cells.

FACS analysis of the cell cycle revealed that SDC4 silencing inhibited myoblast proliferation through decreasing the transition from G1‐ to S‐phase of the cell cycle: the frequency of cells in G1 phase significantly increased, while the frequency of cells in S‐ and G2/M phases significantly decreased following SDC4 silencing (Fig. 5B,C).

Next we analysed the expression of proteins regulating the G1/S transition. The levels of cyclin E and cyclin D1 decreased, and the amount of p21 increased in SDC4 knockdown cells (Fig. 5D) in accordance with the observed G1/S inhibition.

## Discussion

Skeletal muscle is a highly dynamic tissue that can undergo successful regeneration upon injury. SDCs have been reported to play crucial role in muscle development, maintenance and regeneration. The role of SDCs has been shown in muscle development in turkey, mice and *Drosophila* 17, 18, 32, 33, 34. SDC4 is essential during skeletal muscle development and regeneration, and SDC4−/− mice are unable to regenerate damaged muscle 18; however, the underlying mechanisms are poorly understood. Here we have shown that the expression of SDC4 transiently increased during the early stages of notexin‐induced *in vivo* regeneration of soleus muscle, in harmony with the earlier observed transient upregulation of SDC4 mRNA 5. SDC4 is expressed ubiquitously; therefore, it may have originated not only from muscle but from other cell types (e.g. macrophages) on the day 1 of regeneration. However, during *in vitro* myoblast differentiation we found the same expression pattern: SDC4 was highly expressed in proliferating C2C12 myoblast cells, and showed weak expression in differentiated C2C12 myotubes.

Numerous heparan sulfate proteoglycans are expressed in skeletal muscle tissue, including SDCs, glycophosphatidyl‐linked glypicans, extracellular matrix perlecan, agrin or biglycan 35, 36. Here we showed that silencing of SDC4 resulted in the upregulation of heparan sulfate proteoglycans in C2C12 myoblast (SDC1, SDC2, SDC3, glypican‐1, perlecan). Earlier studies described that heparan sulfate chains are involved in myogenesis: inhibition of proteoglycan sulfation by chlorate treatment of either C2C12 cells 37 or intact myofibres 17 affected the proper progression of the myogenic program, and induced the fusion of MM14 myoblasts 38. Heparan sulfate proteoglycans play important role in the regulation of the skeletal muscle satellite cells. The SDCs are considered as co‐receptors for numerous growth factor receptors 7. SDC4 is required for FGF and HGF signalling, and for satellite cell activation 18; SDC3 attenuates FGF and HGF signalling 18, and glypican‐1 enhances differentiation by sequestration of FGF2 39. Loss of SDC3 in satellite cells prevents self‐renewal and rehoming of satellite cells to their niche, maintaining a pool of activated, proliferating cells that largely ameliorate muscular dystrophy in *mdx* mice 40. It has been shown that the binding of FGF2 to its receptor requires prior binding to heparan sulfate 15, and heparan sulfate is required for BMP‐7 signalling 41.

The propeptides (prodomains) of the TGF‐beta superfamily members were shown to target their growth factors to extracellular matrix molecules, for example the propeptide of BMP‐5 interacts with fibrillin‐1 and fibrillin‐2, and the propeptide of myostatin interacts with the glycosaminoglycan (heparan sulfate) chains of perlecan 42. The authors discuss that the TGF‐beta‐like growth factors are targeted to the extracellular matrix (ECM) through specific interactions between propeptides and ECM structural macromolecules 42. Furthermore, perlecan is critical for regulating myostatin signalling 43, and the proteoglycan decorin binds myostatin and influences myostatin signalling 44, 45. Latent TGF‐beta binding proteins (LTBPs) regulate the extracellular availability of latent TGF‐beta. Myostatin forms an inactive complex with LTBP4 leading to a decreased level of active myostatin 46, and LTBP4 contains a heparin‐binding domain 47.

Several members of the TGF‐beta superfamily and their antagonists were shown to bind heparin and heparan sulfates; however, there is no published observation of the binding of myostatin to heparin/heparan sulfates 48. Interestingly, a set of proteins inhibiting myostatin function show affinity towards heparan sulfates: the myostatin propeptide 42, LTBP4 47, or follistatin 49 were described to bind heparane sulfates. Follistatin is an important myostatin antagonist 21, and the myostatin/follistatin complex can bind to heparin, enhancing myostatin cell surface binding 50.

**Figure 6:**
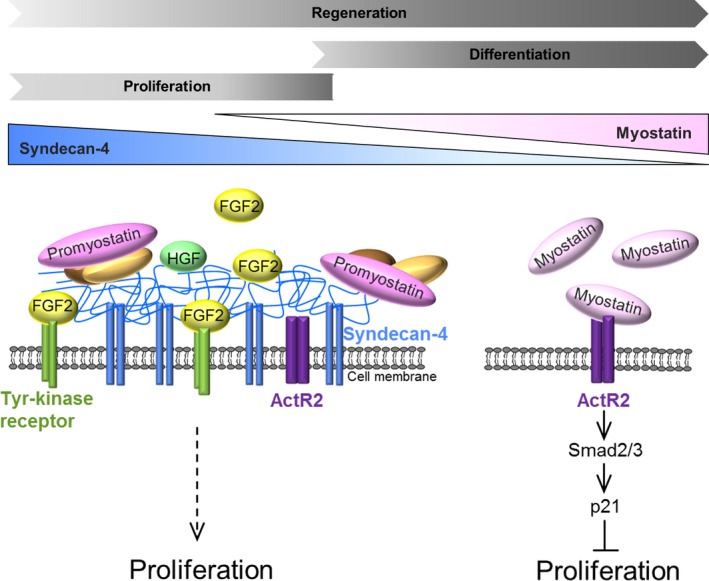
Schematic representation of the role of SDC4 during myoblast proliferation. The level of SDC4 is gradually decreasing during *in vivo* skeletal muscle regeneration, and *in vitro* myoblast differentiation. SDC4 has a complex effect on myoblast proliferation. It can increase myoblast proliferation by enhancing the effect of the proliferative factors (e.g. FGF2, HGF), and by simultaneously decreasing the anti‐proliferative myostatin signalling. The lack of SDC4 increases the level of mature myostatin that can act through its type II Activin receptor (ActRII) to decrease myoblast proliferation. SDC4 may function as a reservoir for the precursor promyostatin; thereby regulating the local bioavailability of mature myostatin by diminishing the formation of the active form (SDC4, syndecan‐4; FGF2, fibroblast growth factor 2; HGF, hepatocyte growth factor).

In this study we found that SDC4, a marker of satellite cells, interacts with the TGF‐beta family member myostatin in a heparan sulfate‐dependent manner. On the basis of the co‐IP experiments, we cannot prove a direct interaction of the heparan sulfate chains with promyostatin, and we cannot exclude the role of other proteins in SDC4‐promyostatin interaction (Fig. 6). Knocking down of SDC4 slightly increased myostatin gene expression. Despite these changes in the transcript level, the amount of promyostatin protein decreased and the level of mature myostatin increased, indicating the enhanced processing of promyostatin protein. Furthermore, SDC4 silencing could also result in changes in promyostatin secretion or protein stability, thus influencing its bioavailability. During skeletal muscle regeneration the level of promyostatin decreased and the level of mature myostatin increased concomitantly, indicating the enhanced proteolytic processing of promyostatin. Interestingly, the level of SDC4 changed in line with the amount of promyostatin, and the high level of SDC4 was associated with low level of mature myostatin. According to this expression pattern and the co‐IP results we can conclude that interaction of SDC4 with promyostatin decreased the cleavage of the latter.

Heparan sulfates can protect the growth factors from proteolytic cleavage 12; therefore, it would be a reasonable hypothesis from our present data that heparan sulfate binding can similarly protect promyostatin from proteolytic activation. Since heparan sulfate chains exhibit promiscuity in binding their respective ligands, we cannot exclude the role of other proteoglycans in promyostatin binding. Here we showed the presence of the other SDC family members, glypican‐1 and perlecan in C2C12 myoblasts; however, SDC4 was the most abundant. It is known that proteolytic maturation of promyostatin can occur extracellularly, beyond the Golgi network 22. We conclude that SDC4 may participate in the maintenance of the extracellular promyostatin pool *via* binding and sequestering promyostatin, thus diminishing the formation of the active, mature myostatin and thereby regulating its local activity.

Heparinase II cleaves heparan sulfate chains to give rise primarily to disaccharides that are unlikely to form stable complexes with promyostatin, allowing its cleavage. Importantly, the mature myostatin was also detected in the digestion buffer collected after heparinase digestion of the immunocomplex; therefore, the intact heparan sulfate chains might have blocked the proteolytic processing of the bound promyostatin, and the heparinase treatment released promyostatin and made it accessible to proteolytic cleavage. The negatively charged heparan sulfate chains show affinity towards electropositive ligands; and the surface‐exposed basic residues of the proteins can provide the binding site for heparan sulfates. This interaction involves the binding of the negatively charged GAG to the amino acid residues lysine and arginine, and can also include protonated histidine residues at low pH values 51. The myostatin itself displays a polar surface potential, with the bottom side facing the cell surface on receptor binding being very electronegative and the top very electropositive 50. Interestingly, a unique continuous electropositive surface is created when myostatin binds the follistatin isoform Fst288, which significantly increases the affinity of follistatin for heparin 50. The mature myostatin domain probably is involved in the binding process to SDC4 given the nature of its electropositive surface, but there is no evidence from our present set of data that the carboxyl terminal mature myostatin is involved in binding to SDC4.

Myostatin was shown to decrease myoblast proliferation by increasing p21 levels 28. In accordance with this result, here we found that SDC4 knockdown increased the amount of myostatin and p21, and decreased the proliferation of the cells. During muscle differentiation the myogenin positive cells remain capable of replicating DNA 52; therefore, the upregulation of p21 will be required to block the cell cycle, which can be the consequence – at least partially – of the increased myostatin signalling.

The inhibition of myostatin signalling by anti‐myostatin antibodies or activin receptor inhibitors seems to be a great challenge to increase muscle mass in case of muscle wasting diseases, for example cancer‐associated cachexia, age‐related sarcopenia or plaster cast immobilization 53. The characterization of signalling pathways playing a role in myoblast proliferation and differentiation is necessary to find new perspectives to improve muscle regeneration following sport injuries, in the case of aging, or muscle dystrophies. Our working model shows that the presence of SDC4 can enhance myoblast proliferation by increasing the effect of proliferative factors (e.g. FGF2, HGF), and by simultaneously decreasing anti‐proliferative myostatin signalling (Fig. 6). SDC4 interacts with promyostatin in a heparan sulfate‐dependent manner and influences the level of mature myostatin. Our results suggest that SDC4 may regulate the local bioavailability of mature myostatin by serving as a reservoir for promyostatin, subsequently inhibiting the formation of active myostatin. Heparan sulfate chains on other proteoglycans are also contributing to the regulation of myostatin but as SDC4 is more highly expressed that the others; therefore, its contribution is likely to be of most importance. Our results can help in better understanding the essential role of SDC4 during skeletal muscle development and regeneration.

## Author contributions

AK‐P conceived and supervised the study; AK‐P designed experiments; AK‐P, TK, KS, AZ and IO performed experiments; LS provided new tools and reagents; AK‐P, KS, TK and FD analysed data; AK‐P, TK and KS prepared figures, AK‐P wrote the manuscript; FD, LS, LP and LD made manuscript revisions.
